# The Influence of Different Treatment Strategies on the Long-Term Prognosis of T1 Stage Esophageal Cancer Patients

**DOI:** 10.3389/fonc.2021.700088

**Published:** 2021-10-14

**Authors:** Liang Pan, Xingyu Liu, Weidong Wang, Linhai Zhu, Wenfeng Yu, Wang Lv, Jian Hu

**Affiliations:** ^1^ Department of Thoracic Surgery, The First Affiliated Hospital, School of Medicine, Zhejiang University, Hangzhou, China; ^2^ Department of General Surgery, The Second Affiliated Hospital, School of Medicine, Zhejiang University, Hangzhou, China; ^3^ Department of Thoracic Surgery, The Hangzhou Chest Hospital, School of Medicine, Zhejiang University, Hangzhou, China

**Keywords:** esophagus cancer, esophagectomy, treatment, prognosis, SEER (surveillance epidemiology and end results) database

## Abstract

**Objective:**

To compare the long-term prognosis effects of non-esophagectomy and esophagectomy on patients with T1 stage esophageal cancer.

**Methods:**

All esophageal cancer patients in the study were included from the National Surveillance Epidemiology and End Results (SEER) database between 2005-2015. These patients were classified into non-esophagectomy group and esophagectomy group according to therapy methods and were compared in terms of esophagus cancer specific survival (ECSS) and overall survival (OS) rates.

**Results:**

A total of 591 patients with T1 stage esophageal cancer were enrolled in this study, including 212 non-esophagectomy patients and 111 esophagectomy patients in the T1a subgroup and 37 non-esophagectomy patients and 140 esophagectomy patients in the T1b subgroup. In all T1 stage esophageal cancer patients, there was no difference in the effect of non-esophagectomy and esophagectomy on postoperative OS, but postoperative ECSS in patients treated with non-esophagectomy was significantly better than those treated with esophagectomy. Cox proportional hazards regression model analysis showed that the risk factors affecting ECSS included race, primary site, tumor size, grade, and AJCC stage but factors affecting OS only include tumor size, grade, and AJCC stage in T1 stage patients. In the subgroup analysis, there was no difference in either ECSS or OS between the non-esophagectomy group and the esophagectomy group in T1a patients. However, in T1b patients, the OS after esophagectomy was considerably better than that of non-esophagectomy.

**Conclusions:**

Non-esophagectomy, including a variety of non-invasive procedures, is a safe and available option for patients with T1a stage esophageal cancer. For some T1b esophageal cancer patients, esophagectomy cannot be replaced at present due to its diagnostic and therapeutic effect on lymph node metastasis.

## Introduction

Esophageal cancer is a malignant tumor mainly originating from the esophageal epithelium and morbidity and mortality of it rank seventh and sixth respectively in the world (1). East Asia has the highest incidence of esophageal cancer in both men and women (1). Esophagectomy has been the standard treatment for esophageal cancer but the extraordinary complications associated with such surgery have not been satisfactory for patients and doctors (2, 3). Recently, several non-invasive treatment modalities are showing great potential in the treatment of early esophageal cancer (4–9). Nevertheless, the impact of these treatments on long-term prognosis differences in patients with early esophageal cancer has not been discussed until now.

On the other hand, we can make accurate T staging for patients with early-stage esophageal cancer, but cannot accurately diagnose lymph node metastasis of these patients before esophagectomy. Many studies have reported the effect of local treatment and surgery on the prognosis of early esophageal cancer patients (10–14). However, there has been no study that compares local treatment with esophagectomy on the long-time prognosis of esophageal cancer patients who have been diagnosed with T stage but do not know whether lymph node metastasis has occurred. And this is the most frequent case in clinical practice.

Given all this, we conducted a retrospective study to compare esophagectomy and non-esophagectomy treatment on long-term survival in T1 stage esophageal cancer patients and determine the risk factors affecting the prognosis of these patients based on the SEER database.

## Materials And Methods

### Study Patients

From 2005 to 2015, there are a total of 11,723 esophagus cancer patients in the SEER database, of whom 591 were enrolled in the study. Inclusion criteria for patients were as follows: 1) histological type of tumor is esophageal carcinoma; 2) patients were first diagnosed with malignant tumor; 3) the diagnosis was made between 2005 and 2015; 4) All enrolled patients were T1 stage esophagus cancer according to the American Joint Committee on Cancer (AJCC) 7th edition TNM stage. Exclusion criteria for patients included: 1) clinical data deficiencies; 2) complicated with other malignant tumors. Detailed information on enrolled patients included demographic records, therapy information, pathology, and prognosis data.

### Group Analysis

All patients listed in the study were divided into the esophagectomy group and the non-esophagectomy group depending on the type of treatment. Non-esophagectomy strategy included local tumor destruction and local tumor excision. Local tumor destruction included electrocautery, cryosurgery and laser. Local tumor excision included polypectomy and excisional biopsy (combination with photodynamic therapy/electrocautery/cryosurgery/laser ablation/laser excision or not).The esophagectomy group included partial esophagectomy, total esophagectomy, and esophagectomy with laryngectomy and/or gastrectomy. T1 stage patients were also divided into T1a group and T1b group to perform subgroup analysis.

### Statistical Analysis

Continuous variables were tested by t test and categorical variables were analyzed using the chi-square test. The Kaplan–Meier curve was used to show the ECSS curve and OS curve. A log-rank test was used to test for significant differences between the esophagectomy group and the non-esophagectomy group. The Cox proportional hazards model that included all variables was used to perform univariate and multivariate analyses. *P*< 0.05 was considered statistically significant. All the data were analyzed using SPSS 19.0 software (SPSS Inc, Chicago, USA) and Graph Pad Prism 5 (Graph Pad Software Inc., La Jolla, USA).

## Results

**Figure 1:**
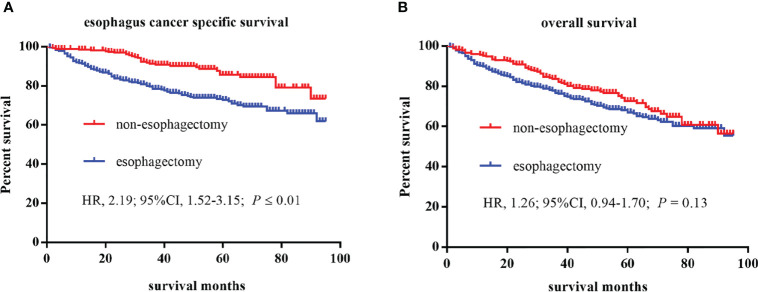
Kaplan–Meier curves of survival estimates for T1 stage esophagus cancer patients who underwent non-esophagectomy and esophagectomy. **(A)**, esophagus cancer specific survival. **(B)**, overall survival. HR, hazard ratio; CI, confidence interval.

A total of 591 patients with esophageal cancer were enrolled in the study, including 275 patients in the non-esophagectomy group and 316 patients in the esophagectomy group as shown in **Table 1**. The follow-up time in the esophagectomy group and the non-esophagectomy group was 49.85 months and 47.55 months respectively. And, there were no significant differences occurred between the two groups. Patients younger than 65 years old in the esophagectomy group were higher than those in the non-esophagectomy group. **Table 1** listed the baseline characteristics, tumor information, and postoperative survival record of all enrolled T1 stage esophagus cancer patients. All variables were statistically significant between the two groups except for age, race, sex, median household income, and location. As shown in **Figure 1**, Kaplan-Meier survival curves of ECSS curve and OS curves for both groups were presented in **Figures 1A, B**, respectively. We can see that ECSS of the non-esophagectomy group was better than the esophagectomy group (HR=2.19; 95%CI, 1.52-3.15; *P* ≤ 0.01) by log-rank test. However, there was no statistically significant difference in OS between the non-esophagectomy group and the esophagectomy group (HR=1.26; 95%CI, 0.94-1.70; *P*=0.13).

**Table 1:** Baseline characteristics of T1 stage esophagus cancer patients with or without esophagectomy between 2005-2015.

		Non-esophagectomy (n = 275)	Esophagectomy (n = 316)	*P*
Age, years (%)	≤65	129 (46.9%)	173 (54.8%)	0.06
	>65	146 (53.1%)	143 (45.2%)	
Race (%)	white	255 (92.7%)	283 (89.6%)	0.18
	others	20 (7.3%)	33 (10.4%)	
Sex (%)	Male	232 (84.4%)	267 (84.5%)	0.97
	Female	43 (15.6%)	49 (15.5%)	
Median household income (%)	<$50000	26 (9.5%)	57 (18.0%)	0.11
	$50000-$ 70000	129 (46.9%)	131 (41.5%)	
	>$70000	120 (43.6%)	128 (40.5%)	
Location (%)	metropolitan	238 (86.6%)	264 (83.5%)	0.31
	nonmetropolitan	37 (13.4%)	52 (16.5%)	
Primary site (%)	Upper	12 (4.4%)	3 (1.0%)	0.01
	Middle	22 (8.0%)	42 (13.3%)	
	Lower	208 (75.6%)	238 (75.3%)	
	Unknown	33 (12.0%)	33 (10.4%)	
Tumor size (cm, %)	≤2	68 (24.7%)	21 (6.7%)	0.01
	>2	207 (75.3%)	295 (93.4%)	
Grade (%)	Grade I-II	126 (45.8%)	165 (52.2%)	0.01
	Grade III-IV	33 (12.0%)	103 (32.6%)	
	Unknown	116 (42.2%)	48 (15.2%)	
Histology (%)	Squamous cell carcinoma	17 (6.2%)	48 (15.2%)	0.01
	Adenocarcinoma	229 (83.3%)	239 (75.6%)	
	others	29 (10.5%)	29 (9.2%)	
AJCC stage (%)	I stage	263 (95.6%)	236 (74.7%)	0.01
	II-IV stage	6 (2.2%)	78 (24.7%)	
	Unknown	6 (2.2%)	2 (0.6%)	
cancer-specific death classification (%)	Alive	242 (88.0%)	228 (72.2%)	0.01
	Dead	33 (12.0%)	88 (27.9%)	
Vital status (%)	Alive	72 (26.2%)	107 (33.9%)	0.04
	Dead	203 (73.8%)	209 (66.1%)	
Survival months, mean (SD)		47.55 (23.2)	49.85 (26.3)	0.26
	T1a stage	212 (77.1%)	111 (35.1%)	
T stage	T1b stage	37 (13.5%)	140 (44.3%)	0.01
	T1NOS	26 (9.4%)	65 (20.6%)	

AJCC, American Joint Committee on Cancer; SD, standard deviation.

Besides, we applied cox proportional hazard regression model to investigate the risk factors affecting the prognosis of patients with T1 stage esophageal cancer. As shown in **Table 2**, we found that race, grade, histology, AJCC stage, and treatment had statistically significant differences for ESCC rate in univariate Cox regression analysis. However, only race, tumor size, and grade had a significant effect on OS rate for T1 stage esophagus cancer patients. In the multivariate Cox proportional hazard regression model, risk factors affecting ESCC of T1 stage esophagus cancer patients were race, AJCC stage and treatment. Similarly, race and AJCC stage also had a significant difference for OS in these patients.

**Table 2:** Cox proportional hazards regression model analysis for ECSS and OS in T1 stage esophagus cancer patients.

		Esophagus cancer-specific survival				Overall survival			
		Univariable		Multivariable		Univariable		Multivariable	
		Hazard Ratio(95% CI)	*P*	Hazard Ratio(95% CI)	*P*	Hazard Ratio(95% CI)	*P*	Hazard Ratio(95% CI)	*P*
Age, years	≤65	1.00 (reference)				1.00 (reference)			
	>65	0.74 (0.52-1.07)	0.11			1.14 (0.85-1.52)	0.40		
Race	white	1.00 (reference)		1.00 (reference)	0.01	1.00 (reference)		1.00 (reference)	0.02
	others	2.17 (1.33-3.54)	0.01	2.16 (1.32-3.54)		0.61 (0.39-0.95)	0.03	1.70 (1.10-2.67)	
Sex	Male	1.00 (reference)				1.00 (reference)			
	Female	1.00 (0.61-1.63)	0.99			1.09 (0.72-1.65)	0.69		
Median household income	<$50000	1.00 (reference)				1.00 (reference)			
	$50000-$ 70000	0.88 (0.53-1.46)	0.62			1.35 (0.87-2.12)	0.18		
	>$70000	0.64 (0.37-1.08)	0.10			1.25 (0.91-1.72)	0.18		
Location	metropolitan	1.00 (reference)				1.00 (reference)			
	nonmetropolitan	1.10 (0.66-1.81)	0.72			0.81 (0.55-1.21)	0.31		
Primary site	Upper	1.00 (reference)				1.00 (reference)			
	Middle	1.13 (0.43-2.98)	0.80			1.55 (0.66-3.61)	0.31		
	Lower	0.46 (0.18-1.12)	0.09			1.46 (0.84-2.56)	0.18		
	Unknown	0.64 (0.24-1.76)	0.39			0.79 (0.51-1.23)	0.30		
Tumor size (cm)	≤2	1.00 (reference)				1.00 (reference)			
	>2	0.84 (0.52-1.37)	0.49			1.51 (1.04-2.20)	0.03		
Grade	Grade I-II	1.00 (reference)				1.00 (reference)			
	Grade III-IV	2.34 (1.59-3.44)	0.01			1.36 (0.91-2.02)	0.03		
	Unknown	0.54 (0.31-0.93)	0.03			2.51 (1.67-3.78)	0.01		
Histology	Squamous cell carcinoma	1.00 (reference)				1.00 (reference)			
	Adenocarcinoma	0.38 (0.24-0.59)	0.01			1.35 (0.77-2.37)	0.30		
	others	0.67 (0.36-1.25)	0.21			0.74 (0.47-1.17)	0.20		
AJCC stage	I stage	1.00 (reference)		1.00 (reference)	0.01	1.00 (reference)		1.00 (reference)	0.01
	II-IV stage	4.00 (2.74-5.84)	0.01	2.35 (1.72-3.20)		0.86 (0.27-2.70)	0.80	1.82 (1.39-2.39)	
Treatment	Non-esophagectomy	1.00 (reference)		1.00 (reference)	0.01	1.00 (reference)			
	esophagectomy	2.24 (1.50-3.35)	0.01	1.93 (1.29-2.89)		0.81 (0.60-1.09)	0.16		

AJCC, American Joint Committee on Cancer; CI, confidence interval.

**Figure 2:**
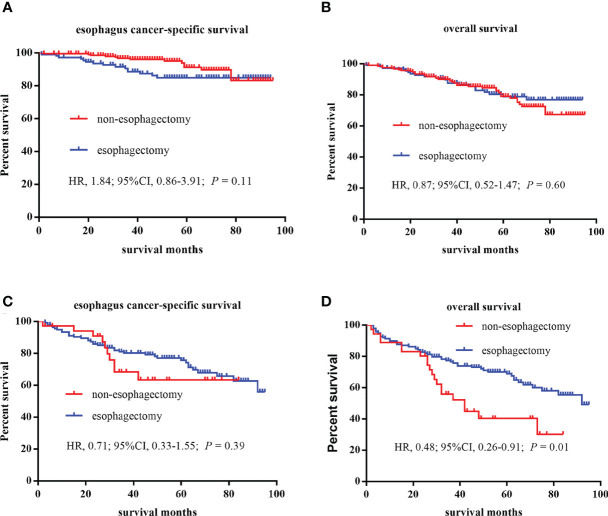
Kaplan–Meier curves of survival estimates for T1a and T1b stage esophagus cancer patients who underwent non-esophagectomy and esophagectomy. **(A)**, esophagus cancer–specific survival for T1a stage patients. **(B)**, overall survival for T1a stage patients. **(C)**, esophagus cancer–specific survival for T1b stage patients. **(D)**, overall survival for T1b stage patients. HR, hazard ratio; CI, confidence interval.

**Table 3** showed the baseline characteristics between the non-esophagectomy group and the esophagectomy group in T1a and T1b stage esophageal cancer patients. Tumor size, grade, and AJCC stage were the baseline variables that differed between the non-esophagectomy group and the esophagectomy group in T1a patients, while in T1b patients, age, tumor size, and AJCC stage were different variables between the non-esophagectomy group and the esophagectomy group. For subgroups of T1 stage patients, different treatments have a different long-term prognosis. As shown in **Figures 2A, B**, there was no significant survival difference between non-esophagectomy group and esophagectomy group, whether ECSS or OS, in T1a stage esophageal cancer patients (HR=1.84; 95%CI, 0.86-3.91; *P*=0.11; HR=0.87; 95%CI, 0.52-1.47; *P*=0.60). For T1b stage patients, there was no significant survival difference between non-esophagectomy group and esophagectomy group for ESCC rate, but not for OS rate (HR=0.71; 95%CI, 0.33-1.55; *P*=0.39; HR=0.48; 95%CI, 0.26-0.91; *P*=0.01) as illustrated in **Figures 2C, D**.

**Table 3:** Baseline characteristics of T1a and T1b stage esophagus cancer patients with or without esophagectomy between 2005-2015.

		T1a esophagus patients			T1b esophagus patients		
		Non-esophagectomy(n=212)	Esophagectomy(n=111)	*P*	Non-esophagectomy(n=37)	Esophagectomy(n=140)	*P*
Age, years (%)	≤65	108 (50.9%)	62 (55.9%)	0.40	10 (27.0%)	75 (53.6%)	0.01
	>65	104 (49.1%)	49 (44.1%)		27 (73.0%)	65 (46.4%)	
Race (%)	white	198 (93.4%)	102 (91.9%)	0.62	35 (94.6%)	124 (88.6%)	0.37
	others	14 (6.6%)	9 (8.1%)		2 (5.4%)	16 (11.4%)	
Sex (%)	Male	184 (86.8%)	96 (86.5%)	0.94	32 (86.5%)	119 (85.0%)	0.82
	Female	28 (13.2%)	15 (13.5%)		5 (13.5%)	21 (15.0%)	
Median household income (%)	<$50000	17 (8.0%)	17 (15.3%)	0.12	5 (13.5%)	30 (21.4%)	0.28
	$50000-$ 70000	101 (47.6%)	47 (42.3%)		20 (54.1%)	56 (40.0%)	
	>$70000	94 (44.3%)	47 (42.3%)		12 (32.4%)	54 (38.6%)	
Location (%)	metropolitan	184 (86.8%)	98 (88.3%)	0.70	31 (83.8%)	121 (86.4%)	0.68
	nonmetropolitan	28 (13.2%)	13 (11.7%)		6 (16.2%)	19 (13.6%)	
Primary site (%)	Upper	6 (2.8%)	0	0.14	2 (5.4%)	2 (1.4%)	0.58
	Middle	11 (5.2%)	8 (7.2%)		5 (13.5%)	20 (14.3%)	
	Lower	172 (81.1%)	85 (76.6%)		27 (73.0%)	109 (77.9%)	
	Unknown	23 (10.8%)	18 (16.2%)		3 (8.1%)	9 (6.4%)	
Tumor size (cm, %)	≤ 2	50 (23.6%)	8 (7.2%)	0.01	7 (18.9%)	1 (0.7%)	0.01
	>2	162 (76.4%)	103 (92.8%)		30 (81.1%)	139 (99.3%)	
Grade (%)	Grade I-II	96 (45.3%)	69 (62.2%)	0.01	21 (56.8%)	70 (50.0%)	0.32
	Grade III-IV	14 (6.6%)	20 (18.0%)		14 (37.8%)	55 (39.3%)	
	Unknown	102 (48.1%)	22 (19.8%)		2 (5.4%)	15 (10.7%)	
Histology (%)	Squamous cell carcinoma	7 (3.3%)	9 (8.1%)	0.15	4 (10.8%)	24 (17.1%)	0.19
	Adenocarcinoma	186 (87.7%)	94 (84.7%)		28 (75.7%)	105 (75.0%)	
	others	19 (9%)	8 (7.2%)		5 (13.5%)	11 (7.9%)	
AJCC stage (%)	I stage	206 (97.2%)	99 (89.2%)	0.01	34 (91.9%)	102 (72.9%)	0.02
	II-IV stage	1 (0.5%)	12 (10.8%)		3 (8.1%)	38 (27.1%)	
	Unknown	5 (2.4%)	0		0	0	
cancer-specific death classification (%)	Alive	197 (92.9%)	96 (86.5%)	0.06	26 (70.3%)	101 (72.1%)	0.82
	Dead	15 (7.1%)	15 (13.5%)		11 (29.7%)	39 (27.9%)	
Vital status (%)	Alive	171 (80.7%)	90 (81.1%)	0.93	16 (43.2%)	90 (35.7%)	0.02
	Dead	41 (19.3%)	21 (18.9%)		21 (56.8%)	50 (64.3%)	
Survival months, mean (SD)		49.9 (22.5)	56.3 (23.8)	0.02	35.8 (22.6)	49.8 (26.5)	0.01

AJCC, American Joint Committee on Cancer; SD, standard deviation.

Then, the cox proportional hazard regression model was performed for T1a and T1b patients respectively. As highlighted in **Table 4**, age, median household income, and AJCC stage were statistically significant risk factors affecting ECSS in T1a stage esophagus cancer patients. Risk factors affecting OS were similar to that of ECSS in these patients, except that age was replaced for tumor size. But for T1b stage esophagus patients, treatment was the only statistically significant factor affecting OS as shown in **Table 5**.

**Table 4:** Cox proportional hazards regression model analysis for ECSS and OS in T1a stage esophagus cancer patients.

		Esophagus cancer specific survival		Overall survival	
		Hazard Ratio (95% CI)	*P*	Hazard Ratio (95% CI)	*P*
Age, years	≤65	1.00 (reference)		1.00 (reference)	
	>65	0.36 (0.15-0.83)	0.02	0.92 (0.56-1.52)	0.74
Race	white	1.00 (reference)		1.00 (reference)	
	others	1.63 (0.49-5.37)	0.43	1.32 (0.53-3.29)	0.56
Sex	Male	1.00 (reference)		1.00 (reference)	
	Female	0.68 (0.21-2.23)	0.52	0.65 (0.28-1.52)	0.32
Median household income	<$50000	1.00 (reference)		1.00 (reference)	
	$50000-$ 70000	0.34 (0.14-0.86)	0.02	0.55 (0.27-1.12)	0.10
	>$70000	0.26 (0.10-0.69)	0.01	0.40 (0.19-0.84)	0.02
Location	metropolitan	1.00 (reference)		1.00 (reference)	
	nonmetropolitan	0.87 (0.26-2.86)	0.81	1.52 (0.77-2.99)	0.23
Primary site	Upper	1.00 (reference)		1.00 (reference)	
	Middle	0.65 (0.06-7.16)	0.72	0.49 (0.08-2.93)	0.43
	Lower	0.47 (0.06-3.48)	0.46	0.53 (0.13-2.20)	0.39
	Unknown	0.99 (0.12-8.07)	0.99	0.75 (0.17-3.41)	0.71
Tumor size (cm)	≤ 2	1.00 (reference)		1.00 (reference)	
	>2	0.62 (0.27-1.46)	0.28	0.54 (0.31-0.96)	0.04
Grade	Grade I-II	1.00 (reference)		1.00 (reference)	
	Grade III-IV	2.50 (1.01-6.19)	0.05	1.41 (0.65-3.10)	0.39
	Unknown	0.81 (0.35-1.88)	0.63	1.18 (0.69-2.01)	0.55
Histology	Squamous cell carcinoma	1.00 (reference)		1.00 (reference)	
	Adenocarcinoma	0.41 (0.12-1.36)	0.14	0.71 (0.26-1.95)	0.50
	others	0.67 (0.15-3.01)	0.60	0.77 (0.22-2.73)	0.68
AJCC stage	I stage	1.00 (reference)		1.00 (reference)	
	II-IV stage	6.26 (2.39-16.39)	0.01	2.76 (1.11-6.89)	0.03
Treatment	Non-esophagectomy	1.00 (reference)		1.00 (reference)	
	esophagectomy	1.67 (0.82-3.43)	0.16	0.85 (0.50-1.44)	0.54

AJCC, American Joint Committee on Cancer; CI, confidence interval.

**Table 5:** Cox proportional hazards regression model analysis for ECSS and OS in T1b stage esophagus cancer patients.

		Esophagus Cancer-specific survival		Overall survival	
		Hazard Ratio (95% CI)	*P*	Hazard Ratio (95% CI)	*P*
Age, years	≤65	1.00 (reference)		1.00 (reference)	
	>65	0.99 (0.57-1.73)	0.98	1.43 (0.89-2.30)	0.14
Race	white	1.00 (reference)		1.00 (reference)	
	others	1.69 (0.79-3.61)	0.18	1.29 (0.64-2.61)	0.48
Sex	Male	1.00 (reference)		1.00 (reference)	
	Female	1.02 (0.46-2.26)	0.97	0.91 (0.45-1.84)	0.80
Median household income	<$50000	1.00 (reference)		1.00 (reference)	
	$50000-$ 70000	1.16 (0.54-2.49)	0.71	1.03 (0.54-1.98)	0.92
	>$70000	0.97 (0.43-2.17)	0.94	1.07 (0.55-2.07)	0.85
Location	metropolitan	1.00 (reference)		1.00 (reference)	
	nonmetropolitan	0.44 (0.14-1.43)	0.17	0.63 (0.27-1.47)	0.29
Primary site	Upper	1.00 (reference)		1.00 (reference)	
	Middle	1.59 (0.20-12.66)	0.66	1.00 (0.22-4.50)	0.99
	Lower	0.86 (0.12-6.39)	0.89	0.60 (0.15-2.49)	0.48
	Unknown	0.46 (0.04-5.11)	0.53	0.45 (0.08-2.47)	0.36
Tumor size (cm)	≤ 2	1.00 (reference)		1.00 (reference)	
	>2	0.79 (0.19-3.27)	0.74	0.44 (0.18-1.09)	0.08
Grade	Grade I-II	1.00 (reference)		1.00 (reference)	
	Grade III-IV	1.05 (0.59-1.86)	0.88	1.11 (0.69-1.80)	0.67
	Unknown	0.51 (0.15-1.68)	0.27	0.48 (0.17-1.36)	0.17
Histology	Squamous cell carcinoma	1.00 (reference)		1.00 (reference)	
	Adenocarcinoma	0.64 (0.31-1.29)	0.21	0.89 (0.46-1.70)	0.72
	others	1.14 (0.43-3.00)	0.79	1.30 (0.54-3.15)	0.56
AJCC stage	I stage	1.00 (reference)		1.00 (reference)	
	II-IV stage	1.49 (0.81-2.74)	0.20	1.17 (0.69-2.00)	0.57
Treatment	Non-esophagectomy	1.00 (reference)		1.00 (reference)	
	esophagectomy	0.70 (0.35-1.37)	0.29	0.47 (0.28-0.78)	0.01

AJCC, American Joint Committee on Cancer; CI, confidence interval.

## Discussion

With the popularization of digestive tract tumor screening, especially the increasingly standardized endoscopic monitoring of Barret’s esophagus, more and more early stage esophageal cancer have been diagnosed in both East and West (15, 16). On the other hand, a variety of new minimally invasive treatments for early esophageal cancer have been increasingly attempted. McCaughan et al. first applied photodynamic therapy to esophageal cancer as a sensitizer before radiation therapy in 1984 (17). Mondragón et al. firstly reported that esophageal endoscopic resection was safe and available for esophageal leiomyoma using the submucosal tunneling technique (18). Since then, various studies on minimally invasive treatment for early esophageal cancer have emerged one after another, but the application of non-esophagectomy in the treatment of early esophageal cancer has been controversial especially for the subgroup of T1 stage esophagus cancer patients (19–24). In this study, we showed the effects of both non-esophagectomy and esophagectomy on the long-term survival of T1 stage esophageal cancer. As you can see from the results, the long-term survival of patients with T1a stage esophageal cancer after non-esophagectomy treatment was not inferior to that after esophagectomy either ECSS or OS. However, for patients with T1b stage esophageal cancer, the effect of esophagectomy on OS is far superior to that of patients treated with non-esophagectomy.

Some studies have tried to evaluate the possibility of non-esophagectomy in the treatment of T1 stage esophageal cancer patients (25–29). Semenkovich et al. showed that endoscopic therapy was an alternative treatment of esophagectomy for T1a esophagus cancer patients (14). Prasad et al. revealed that OS in T1a stage esophagus cancer patients treated endoscopically were comparable with that of patients treated surgically (30). George et al. conducted a systematic review of the effects of endoscopy and surgery on T1 stage esophageal cancer patients, which indicated that the T1b esophagus should be surgical resection (31). We acquired the same results in this study which based on long-term follow up. Our results also showed that non-esophagectomy was a safe and available option for T1a stage esophagus cancer patients, but not adapted to T1b stage esophagus cancer patients, at least for all T1b stage patients. So, these results strongly suggest the rationality of non-esophagectomy in the treatment of T1a stage esophageal cancer patients and the difference of non-esophagectomy and esophagectomy in the prognosis of T1b stage esophagus cancer.

The results of subgroup analysis showed that the prognosis of non-esophagectomy for T1b stage esophageal cancer patients was worse than that of esophagectomy, which implied the diversity of patients with T1b stage esophageal cancer. It was reported that T1b stage esophageal cancer was more deeply invasive and had a greater risk of lymph node metastasis compared with T1a stage esophageal cancer (25, 32). Moreover, the results of our Cox proportional hazard model analysis also suggested that tumor stage was always an important influencing factor for the long-term prognosis of patients with T1 esophageal cancer, regardless of ECSS or OS. Therefore, it is necessary to classify T1b stage esophageal cancer and evaluate the effects of non-esophagectomy and esophagectomy in subgroups of T1b stage esophageal cancer patients. Previous studies have reported that T1 stage esophageal cancer can be divided into subgroups according to the depth of invasion and predict lymph node metastasis and guide treatment (33, 34). However, lymph node metastasis of early esophageal cancer is a process associated with many factors, including tumor size, grade, pathological type, and so on. Therefore, a new staging system to guide the treatment of T1b esophageal cancer will be necessary.

Of course, there are some limitations to this study. First of all, this study has some inevitable bias since the nature of a retrospective study. We expect a multicenter prospective study to further reveal the effect of different treatments on the long-term prognosis of early esophageal cancer patients. Secondly, since the data of postoperative adjuvant therapy for patients with T1 stage esophageal cancer were not included, we could not rule out the possibility that the influence of postoperative adjuvant therapy on the prognosis of these patients. Last but not least, patients with T1b stage esophageal cancer are a very heterogeneous population in term of TNM stage. But due to the limitation of T1b sample size, we did not conduct subgroup analysis on these patients. The sample size contained in the present study is not large, which may cause certain biases to the statistical results.

In conclusion, non-esophagectomy is a treatment option that is not inferior to esophagectomy at least in patients with T1a esophageal cancer. Esophagectomy remains the choice for some patients with T1b stage esophageal cancer, but treatment methods should be selected according to the different conditions of patients.

## Data Availability Statement

Publicly available datasets were analyzed in this study. This data can be found here: SEER (https://seer.cancer.gov/data/) database.

## Author Contributions 

LP: Conceptualization, Formal Analysis, Data curation, Software, Visualization, Writing - original draft. XL: Conceptualization, Investigation, Methodology, Writing - original draft. WW: Investigation, Software. LZ and WY: Methodology, Visualization. WL: Investigation, Visualization. JH: Conceptualization, Data curation, Funding acquisition, Project administration, Resources, Supervision, Validation, Writing- review & editing. All authors contributed to the article and approved the submitted version.

## Funding

Key Project of Zhejiang Province Science and Technology Plan (2020C03058), Zhejiang Province Key Discipline of Traditional Chinese Medicine (2017-XK-A33), Zhejiang Provincial Medical and Science and Technology Plan (2016KYB258).

## Conflict of Interest

The authors declare that the research was conducted in the absence of any commercial or financial relationships that could be construed as a potential conflict of interest.

## Publisher’s Note

All claims expressed in this article are solely those of the authors and do not necessarily represent those of their affiliated organizations, or those of the publisher, the editors and the reviewers. Any product that may be evaluated in this article, or claim that may be made by its manufacturer, is not guaranteed or endorsed by the publisher.
